# CovidNeuroOnc: A UK multicenter, prospective cohort study of the impact of the COVID-19 pandemic on the neuro-oncology service

**DOI:** 10.1093/noajnl/vdab014

**Published:** 2021-01-28

**Authors:** Daniel M Fountain, Rory J Piper, Michael T C Poon, Georgios Solomou, Paul M Brennan, Yasir A Chowdhury, Francesca Colombo, Tarek Elmoslemany, Frederick G Ewbank, Paul L Grundy, Md T Hasan, Molly Hilling, Peter J Hutchinson, Konstantina Karabatsou, Angelos G Kolias, Nathan J McSorley, Christopher P Millward, Isaac Phang, Puneet Plaha, Stephen J Price, Ola Rominiyi, William Sage, Syed Shumon, Ines L Silva, Stuart J Smith, Surash Surash, Simon Thomson, Jun Y Lau, Colin Watts, Michael D Jenkinson, British Neurosurgical Trainee Research Collaborative (BNTRC), Yahia Al-Tamimi, Andrew F Alalade, Erminia Albanese, Matthew Bailey, Andrew R Brodbelt, Anthony Chalmers, Huan Wee Chan, David J Coope, Sarah Cundliffe, Pietro I D'Urso, Helen Entwistle, Rhiannon M Evans, Rebecca Fielding, Christos Gkolemis, Charlotte Hammerbeck-Ward, D Sanjeeva Jeyaretna, Andrew T King, Raphael M Laurente, James Leggate, Rachel Lewis, Jillian Maclean, Catherine McBain, Grainne S McKenna, Elizabeth Molloy, Omar N Pathmanaban, Pradnya Patkar, James Powell, Scott A Rutherford, Thomas Santarius, Saurabh Sinha, Murugan Sitaraman, Anna Solth, Bhaskar Thakur, Andrea Wadeson, Victoria Wykes, Muhammed R Zafar

**Affiliations:** Manchester Centre for Clinical Neurosciences, Salford Royal NHS Foundation Trust, Salford, UK; University of Manchester, Manchester, UK; Department of Neurosurgery, John Radcliffe Hospital, Oxford, UK; Centre for Medical Informatics, Usher Institute, University of Edinburgh, Edinburgh, UK; School of Medicine, Keele University, Staffordshire, UK; Centre for Clinical Brain Sciences, University of Edinburgh, Edinburgh, UK; Department of Neurosurgery, Queen Elizabeth Hospital Birmingham, University Hospitals Birmingham NHS Foundation Trust, Birmingham, UK; Manchester Centre for Clinical Neurosciences, Salford Royal NHS Foundation Trust, Salford, UK; University of Manchester, Manchester, UK; Department of Neurosurgery, The Walton Centre NHS Foundation Trust, Liverpool, UK; University of Liverpool, Liverpool, UK; Department of Neurosurgery, University Hospital Southampton NHS Foundation Trust, Southampton, UK; Department of Neurosurgery, University Hospital Southampton NHS Foundation Trust, Southampton, UK; Manchester Centre for Clinical Neurosciences, Salford Royal NHS Foundation Trust, Salford, UK; University of Manchester, Manchester, UK; Department of Neurosurgery, The Royal London Hospital, Barts Health NHS Trust, London, UK; Division of Neurosurgery, Department of Clinical Neurosciences, Addenbrooke's Hospital and University of Cambridge, Cambridge, UK; Manchester Centre for Clinical Neurosciences, Salford Royal NHS Foundation Trust, Salford, UK; University of Manchester, Manchester, UK; Division of Neurosurgery, Department of Clinical Neurosciences, Addenbrooke's Hospital and University of Cambridge, Cambridge, UK; Department of Neurosurgery, Ninewells Hospital, Dundee, UK; Department of Neurosurgery, The Walton Centre NHS Foundation Trust, Liverpool, UK; Department of Neurosurgery, Lancashire Teaching Hospitals NHS Foundation Trust, Preston, UK; Department of Neurosurgery, John Radcliffe Hospital, Oxford, UK; Division of Neurosurgery, Department of Clinical Neurosciences, Addenbrooke's Hospital and University of Cambridge, Cambridge, UK; Department of Neurosurgery, Sheffield Teaching Hospitals NHS Foundation Trust, Sheffield, UK; Department of Neurosurgery, School of Medicine, Queen's Medical Centre, University of Nottingham, Nottingham, UK; Department of Neurosurgery, Royal Victoria Infirmary, Newcastle-upon-Tyne, UK; Department of Neurosurgery, The Royal London Hospital, Barts Health NHS Trust, London, UK; Department of Neurosurgery, School of Medicine, Queen's Medical Centre, University of Nottingham, Nottingham, UK; Department of Neurosurgery, Royal Victoria Infirmary, Newcastle-upon-Tyne, UK; Department of Neurosurgery, Leeds General Infirmary, Leeds, UK; Department of Neurosurgery, John Radcliffe Hospital, Oxford, UK; Department of Neurosurgery, Queen Elizabeth Hospital Birmingham, University Hospitals Birmingham NHS Foundation Trust, Birmingham, UK; Institute of Cancer and Genome Sciences, University of Birmingham, Birmingham, UK; Department of Neurosurgery, The Walton Centre NHS Foundation Trust, Liverpool, UK; University of Liverpool, Liverpool, UK

**Keywords:** brain tumor, COVID-19, intracranial tumor, neuro-oncology, SARS-CoV-2

## Abstract

**Background:**

The COVID-19 pandemic has profoundly affected cancer services. Our objective was to determine the effect of the COVID-19 pandemic on decision making and the resulting outcomes for patients with newly diagnosed or recurrent intracranial tumors.

**Methods:**

We performed a multicenter prospective study of all adult patients discussed in weekly neuro-oncology and skull base multidisciplinary team meetings who had a newly diagnosed or recurrent intracranial (excluding pituitary) tumor between 01 April and 31 May 2020. All patients had at least 30-day follow-up data. Descriptive statistical reporting was used.

**Results:**

There were 1357 referrals for newly diagnosed or recurrent intracranial tumors across 15 neuro-oncology centers. Of centers with all intracranial tumors, a change in initial management was reported in 8.6% of cases (*n* = 104/1210). Decisions to change the management plan reduced over time from a peak of 19% referrals at the start of the study to 0% by the end of the study period. Changes in management were reported in 16% (*n* = 75/466) of cases previously recommended for surgery and 28% of cases previously recommended for chemotherapy (*n* = 20/72). The reported SARS-CoV-2 infection rate was similar in surgical and non-surgical patients (2.6% vs. 2.4%, *P* > .9).

**Conclusions:**

Disruption to neuro-oncology services in the UK caused by the COVID-19 pandemic was most marked in the first month, affecting all diagnoses. Patients considered for chemotherapy were most affected. In those recommended surgical treatment this was successfully completed. Longer-term outcome data will evaluate oncological treatments received by these patients and overall survival.

Key Points

- 8.6% of patients with brain tumors received changes in treatment due to COVID-19.
- Overall rates decreased from 19% at the start of April 2020 to 0% by end May 2020.
- SARS-CoV-2 infection was not higher in patients undergoing surgery.

**Importance of the Study**

This study reports the effect that the COVID-19 pandemic has imposed on clinical decision making within the UK neuro-oncology service. In 1210 consecutive patients with an intracranial tumor referred to their local neuro-oncology or skull base multidisciplinary team during the COVID-19 pandemic, 8.6% were recommended a management plan different to usual care. This affected 28% of patients who would have been usually offered chemotherapy and 16% of patients who would have been usually offered surgery. This study showed that the deviation from usual care was at its peak in April 2020 (19%) and decreased to 0% change by the end of the study period (May 2020). SARS-CoV-2 infection was not higher in patients undergoing surgery. Decisions relating to management of patients during the COVID-19 pandemic will be multifactorial and center-specific with considerations to the presenting symptoms of the patient, local case incidence, and healthcare resource availability.

The severe acute respiratory syndrome coronavirus 2 (SARS-CoV-2) (COVID-19) pandemic has caused an unprecedented impact on the UK *National Health Service* (NHS). Major restrictions on resources and capacity have affected provision of both medical and surgical cancer therapies.^1^ Cancer Research UK documented a 60% reduction in cancer surgery and an international study by the *CovidSurg Collaborative* reported that an estimated 2.3 million elective cancer cases had been cancelled worldwide.^2,3^ Delays to cancer surgery can impact on overall survival. A three-month delay across all stage 1 to 3 cancers is estimated to cause >4700 attributable deaths per year in England alone.^4^

Delaying surgical treatment of a brain tumor can lead to irreversible neurological impairment and be rapidly life-threatening because of the risk of raised intracranial pressure and coma. Two international reports from over 90 countries, reported cancellation rates of up to 57.5% for neurosurgical operations and clinics across the globe.^5,6^ Furthermore, there are considerable risks from surgery for patients with SARS-CoV-2 infection. An international pan-specialty study by the *CovidSurg Collaborative* showed that in 1128 patients with a perioperative SARS-CoV-2 infection, the mortality rate was 24% and 51% had pulmonary complications.^7^

Guidance set out by the *British Neuro-Oncology Society* (BNOS) and the *Society of British Neurological Surgeons* (SBNS) on the 19th March 2020 made several recommendations for surgical and oncological practice during the COVID-19 pandemic,^8^ including giving high surgical and oncology priority to patients with:

- Malignant gliomas suitable for surgery and adjuvant therapies.
- Posterior fossa tumors causing symptoms or hydrocephalus.
- Meningiomas causing major mass effect or neurological deficit.
- Brain metastases suitable for surgery and supratentorial, or suitable for stereotactic radiosurgery or whole brain radiotherapy.

Conversely, low surgical and oncology priority were designated to patients with:

- Low-grade glioma where active monitoring is a reasonable option.
- Skull base tumors where the patient was already planned for elective surgery.
- Radiotherapy for atypical/recurrent meningioma.

Guidelines regarding the overall surgical and adjuvant therapies for high-grade gliomas have also been published by an international consensus group.^9^

The COVID-19 pandemic presented several problems including how to maintain a safe surgical neuro-oncology service, the risks posed to patients undergoing treatment, and how the decisions made by the neuro-oncology multidisciplinary teams (MDT, a team of professionals including neuro-oncologists, radiologists, neuropathologists, specialist nurses, and neurosurgeons facilitating shared decision making between specialties—known as tumor board in North America) were affected. We therefore conducted the CovidNeuroOnc multicenter, prospective cohort study to assess the impact of the COVID-19 pandemic on the UK neuro-oncology service for patients with newly diagnosed or recurrent brain tumors.

## Methods

### Study Design

CovidNeuroOnc is a national, multicenter, prospective observational study in the UK. We invited all adult neurosurgical units in the UK to collaborate on this study and 15 of 32 participated. The study was designed and delivered by the *British Neurosurgical Trainee Research Collaborative (BNTRC)*^10^ and the *Academic Committee of the Society of British Neurological Surgeons (SBNS)*.

### Patient Identification

Consecutive patients were identified from weekly neuro-oncology and skull base MDT (tumor board) meetings between 1st April and 31st May 2020 in participating units. All patients aged ≥16 years were included if they were found to have a newly diagnosed or recurrent intracranial tumor (including low-grade glioma, high-grade glioma, primary central nervous system lymphoma, meningioma, vestibular schwannoma, or metastases) based on either computed tomography (CT) or magnetic resonance imaging (MRI). Pituitary tumors were excluded from this study due to the possible endocrinological management and the separate MDT management of these tumors.

### Data Collection

De-identified data were collected using a secure, online data collection tool (www.castoredc.com). Each local collaborator was given a unique account to facilitate an accurate audit trail. Data fields included: age, sex, *Eastern Cooperative Oncology Group* (ECOG) performance status, date of MDT, and radiological diagnosis. We asked collaborators to record: (i) the “pre-COVID-19” MDT decision (the hypothetical decision of “usual” first line management that the MDT would have made without the influence of COVID-19), and (ii) the “post-COVID-19” MDT decision, which is the first line management offered during the COVID-19 period. Sites recorded a single management option from the following: surgery (biopsy or resection), chemotherapy, fractionated radiotherapy, stereotactic radiosurgery, active monitoring (watch and wait), no treatment required or best supportive care. A decision to delay or defer treatment was included when asking sites for “post-COVID-19” MDT decisions. Data were also collected on the types and dates of treatments administered (surgery, chemotherapy, radiotherapy, radiosurgery, active monitoring), and date of confirmed COVID-19 status (if applicable). Extent of resection was confirmed on postoperative MRI where it occurred over the course of the study period. Date of death was also recorded for patients with suspected high-grade glioma based on MRI or confirmed high-grade glioma after surgery. SARS-CoV-2 infection was determined either by viral RNA detection (nose and throat swab) or by CT chest imaging as per the diagnostic process during the study period. Data collection was finalized on the 30th of June 2020 to allow 30-day follow-up following the index MDT within the study period. All participating units attained local departmental approval as a service evaluation prior to anonymized data collection and submission such that individual consent was not required. Additional daily COVID-19 confirmed cases were retrieved for temporal analysis from the UK government.^11^

### Objectives

The primary objective was to determine whether the COVID-19 pandemic changed the management of patients with either newly diagnosed or recurrent intracranial tumors, compared to usual care. Secondary objectives were to determine (i) how many patients did not receive surgery, despite this being the MDT recommendation, (ii) how many patients contracted a SARS-CoV-2 infection, and (iii) how many patients with high-grade glioma died during the study period up to the first data lock on June 30, 2020.

### Statistical Analysis

Categorical variables were reported as percentages. Continuous variables were reported as median and interquartile range (IQR) or mean and standard deviation based on tests for normality with the Shapiro–Wilk test. Univariable categorical statistical tests were performed with Chi-square testing unless small samples sizes where Fisher’s exact testing was utilized. Odds ratios and 95% confidence intervals were computed using the Wald test. A threshold *P*-value of <.05 was set to denote statistical significance. All analyses, tables, and graphics including Sankey diagrams were completed using the *tidyverse*, *gtsummary*, *epitools*, *RColorBrewer*, and *riverplot* packages in R v 3.6.0.^12–16^

## Results

There were 1357 consecutive referrals for newly diagnosed or recurrent intracranial tumors across 15 regional neurosurgical units in the United Kingdom between 1st April and 31st May 2020. Fourteen units provided data on all intracranial tumors, while one unit provided data on malignant gliomas only (*n* = 147). Data from this unit were excluded from total cohort summative statistic and included for specific analysis of malignant gliomas to optimize external validity. Descriptive statistics for the remaining 1210 referrals are presented in Tables 1 and 2. The majority of referrals were for newly diagnosed intracranial tumors (*n* = 950, 79%) and included patients aged 50–80 years old (*n* = 858, 71%) who were ECOG performance status 0 or 1 (*n* = 862/1210, 71%). The most common referral of a new intracranial tumor was for metastasis (*n* = 344, 36%) or high-grade glioma (*n* = 295, 31%), whereas the most common recurrence was for glioma (*n* = 130/260, 50%, Table 2).

**Table 1.** Descriptive Statistics Stratified by Presentation (*n* = 1210)

Characteristic	Overall, *n* = 1210	New Diagnosis, *n* = 950^a^	Recurrence, *n* = 260^a^	*P*-value^b^
Age				<0.001
16–19	5 (0.4%)	4 (0.4%)	1 (0.4%)	
20–29	35 (2.9%)	26 (2.7%)	9 (3.5%)	
30–39	66 (5.5%)	37 (3.9%)	29 (11%)	
40–49	111 (9.2%)	73 (7.7%)	38 (15%)	
50–59	256 (21%)	190 (20%)	66 (25%)	
60–69	307 (25%)	253 (27%)	54 (21%)	
70–79	295 (24%)	245 (26%)	50 (19%)	
80–89	123 (10%)	110 (12%)	13 (5.0%)	
90+	12 (1.0%)	12 (1.3%)	0 (0%)	
Sex				0.2
Female	619 (51%)	495 (52%)	124 (48%)	
Male	591 (49%)	455 (48%)	136 (52%)	
ECOG				0.11
0	466 (39%)	376 (40%)	90 (35%)	
1	396 (33%)	294 (31%)	102 (40%)	
2	204 (17%)	162 (17%)	42 (16%)	
3	102 (8.5%)	84 (8.9%)	18 (7.0%)	
4	29 (2.4%)	25 (2.7%)	4 (1.6%)	

^a^Statistics presented: *n* (%).

^b^Statistical tests performed: chi-square test of independence.

**Table 2.** Descriptive Statistics of Most Likely Diagnosis Based on Radiological Imaging for Newly Diagnosed Intracranial Tumors and Original Histopathology for Recurrent Tumors (*n* = 1210)

New Diagnosis—Radiological Diagnosis	*n* = 950^a^
High-grade glioma	295 (31%)
Low-grade glioma	60 (6.3%)
Meningioma	157 (17%)
Metastasis	344 (36%)
Other^b^	41 (4.3%)
Primary CNS lymphoma	40 (4.2%)
Vestibular schwannoma	11 (1.2%)
Missing	2 (0.2%)
Recurrence—Original Histopathology	*n* = 260^a^
Glioma	130 (50%)
Meningioma	27 (10%)
Metastasis	72 (28%)
Other^c^	22 (8.5%)
Primary CNS lymphoma	3 (1.2%)
Vestibular schwannoma	6 (2.3%)

^a^Statistics presented: *n* (%).

^b^“Other” included chordoma, pineal tumor, ependymoma, ganglioglioma, and pineocytoma.

^c^“Other” included chordoma, chondrosarcoma, choroid plexus papilloma, ependymoma, ganglioglioma, and medulloblastoma.

### Primary Outcome

**Figure 1.**
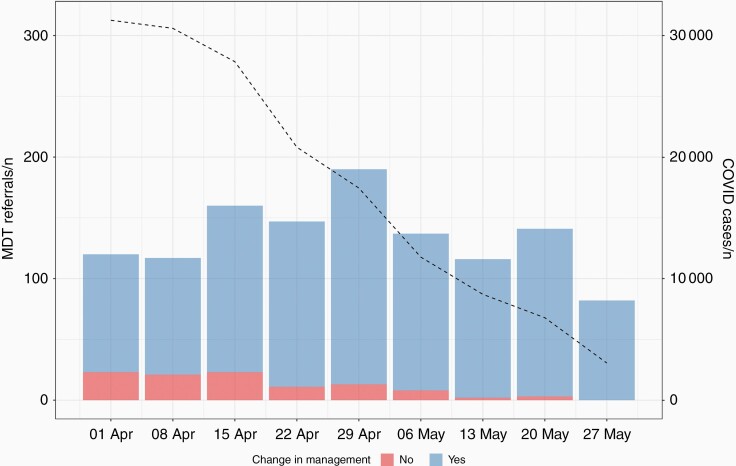
MDT referrals by week stratified by primary outcome with overlay of weekly COVID-19 cases in the United Kingdom (*n* = 1210, note week commencing 27th May was 5 days).

Overall, 8.6% of cases had a documented change in MDT decision compared to usual care (*n* = 104/1210). Figure 1 shows the trends in weekly COVID-19 cases and number of referrals to the neuro-oncology MDT stratified by change in management. Changes in MDT decision were more likely in recurrent than newly diagnosed tumors (OR 1.8 95% CI 1.2–2.8, *P* = .010). Over the study period, there was a significant reduction in the number of patients where COVID-19 resulted in a change in management plan at the MDT. In the first week of the study, a change in MDT decision was seen in 19% (*n* = 23/120) of referrals, and this reduced to 0% (*n* = 0/82) by the end of May 2020. The majority of referrals with a change in MDT decision occurred in the first 4 weeks of the study period, which corresponded to the peak of the pandemic in the UK (*n* = 78/104, 75%).

**Figure 2.**
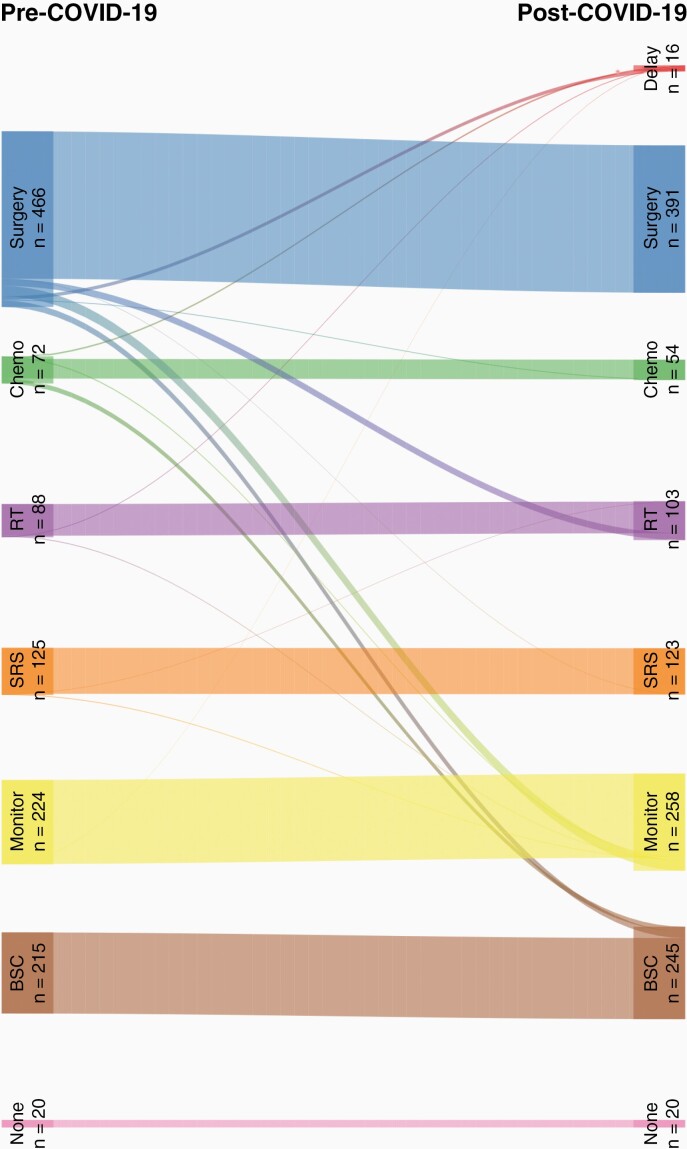
Sankey diagram of change in management decision as a result of the COVID-19 pandemic (*n* = 1210). Delay = delay or defer treatment, Chemo = chemotherapy, RT = radiotherapy, SRS = stereotactic radiosurgery, Monitor = interval monitoring, None = no treatment required, BSC = best supportive care.

The pre-COVID-19 and post-COVID-19 MDT decision are depicted in the Sankey diagram in Figure 2. The most common pre-COVID-19 management in all cases was surgery (*n* = 466, 39%). While a small proportion of patients were subject to a delay or deferral of treatment (*n* = 16, 1%), there was a larger proportion of patients where the MDT decision changed from surgical intervention. Of the 466 patients considered for surgery in pre-COVID-19 “usual care” decisions, 75 (16%) patients were instead offered alternative management plans including active monitoring (*n* = 28, 37%), radiotherapy (*n* = 18, 24%), best supportive care (*n* = 17, 23%), and a delay in treatment (*n* = 9, 12%). Of the 72 patients considered for chemotherapy in pre-COVID-19 decisions, 20 (28%) were subsequently offered alternatives, most commonly best supportive care (*n* = 13, 65%).

Given the large proportion of patients who would have been offered no treatment or best supportive care in a pre-COVID-19 situation (*n* = 235, 19%), these were excluded for the purpose of identifying factors resulting in a change in management as a result of COVID-19. Comparative descriptive statistics of the remaining 975 referrals are shown in Table 3. There was no significant difference in age, sex, or ECOG, but patients presenting with a recurrence and in particular recurrent glioma were more likely to have a change in management plans (OR 3.3 95% CI 1.5–7.9, *P* = .003). Patients referred to the MDT with a suspected SARS-CoV-2 infection at the time of MRI diagnosis were no more likely to be offered a change in management plan (*P* = .4).

**Table 3.** Descriptive Statistics of Cohort Stratified by Whether MDT Recommended Management Changed as a Result of COVID-19 (*n* = 975)

Characteristic	No, *n* = 871^a^	Yes, *n* = 104^a^	*P*-value^b^
Age			.889
16–19	4 (0.5%)	1 (1.0%)	
20–29	31 (3.6%)	4 (3.8%)	
30–39	58 (6.7%)	6 (5.8%)	
40–49	94 (11%)	10 (9.6%)	
50–59	210 (24%)	25 (24%)	
60–69	243 (28%)	24 (23%)	
70–79	190 (22%)	27 (26%)	
80–89	41 (4.7%)	7 (6.7%)	
Sex			>.9
Female	445 (51%)	52 (50%)	
Male	426 (49%)	52 (50%)	
ECOG			.4
0	388 (45%)	43 (42%)	
1	317 (37%)	33 (32%)	
2	129 (15%)	22 (22%)	
3	24 (2.8%)	4 (3.9%)	
4	4 (0.5%)	0 (0%)	
Presentation			.042
New diagnosis	677 (78%)	71 (68%)	
Recurrence	194 (22%)	33 (32%)	
New diagnosis—radiological diagnosis			.085
High-grade glioma	193 (29%)	23 (32%)	
Low-grade glioma	48 (7.1%)	10 (14%)	
Meningioma	124 (18%)	16 (23%)	
Metastasis	238 (35%)	14 (20%)	
Other	34 (5.0%)	2 (2.8%)	
Primary CNS lymphoma	30 (4.4%)	5 (7.0%)	
Vestibular schwannoma	9 (1.3%)	1 (1.4%)	
Recurrence—original histopathology			.002
Glioma	86 (44%)	24 (73%)	
Meningioma	22 (11%)	3 (9.1%)	
Metastasis	62 (32%)	1 (3.0%)	
Other	17 (8.8%)	3 (9.1%)	
Primary CNS lymphoma	2 (1.0%)	1 (3.0%)	
Vestibular schwannoma	5 (2.6%)	1 (3.0%)	
SARS–CoV-2 suspected at time of MRI diagnosis	16 (1.8%)	3 (2.9%)	.4

^a^Statistics presented: *n* (%).

^b^ Statistical tests performed: chi-square test of independence; Fisher's exact test.

### Surgical Treatment

Descriptive statistics of all 354 patients who underwent surgery up to 30th June 2020 are presented in the Supplementary Material (Supplementary Table S1). Of the 391 patients with a plan for surgery following the MDT, 345 (88%) were recorded to have undergone surgery, with 368 operations performed in total. The majority of surgery performed was for glioma (newly diagnosed *n* = 180/313, 58%, recurrent *n* = 21/41, 51%) and metastasis (newly diagnosed *n* = 72/313, 23%, recurrent *n* = 10/41, 24%). Surgical and histopathological data is provided in the Supplementary Material (Supplementary Table S2). No patient with an MDT plan for resection underwent a biopsy subsequently. Gross-total resection was achieved in 69% of cases where resection was planned in the MDT (*n* = 180/261).

### Management of High-grade Glioma

**Figure 3.**
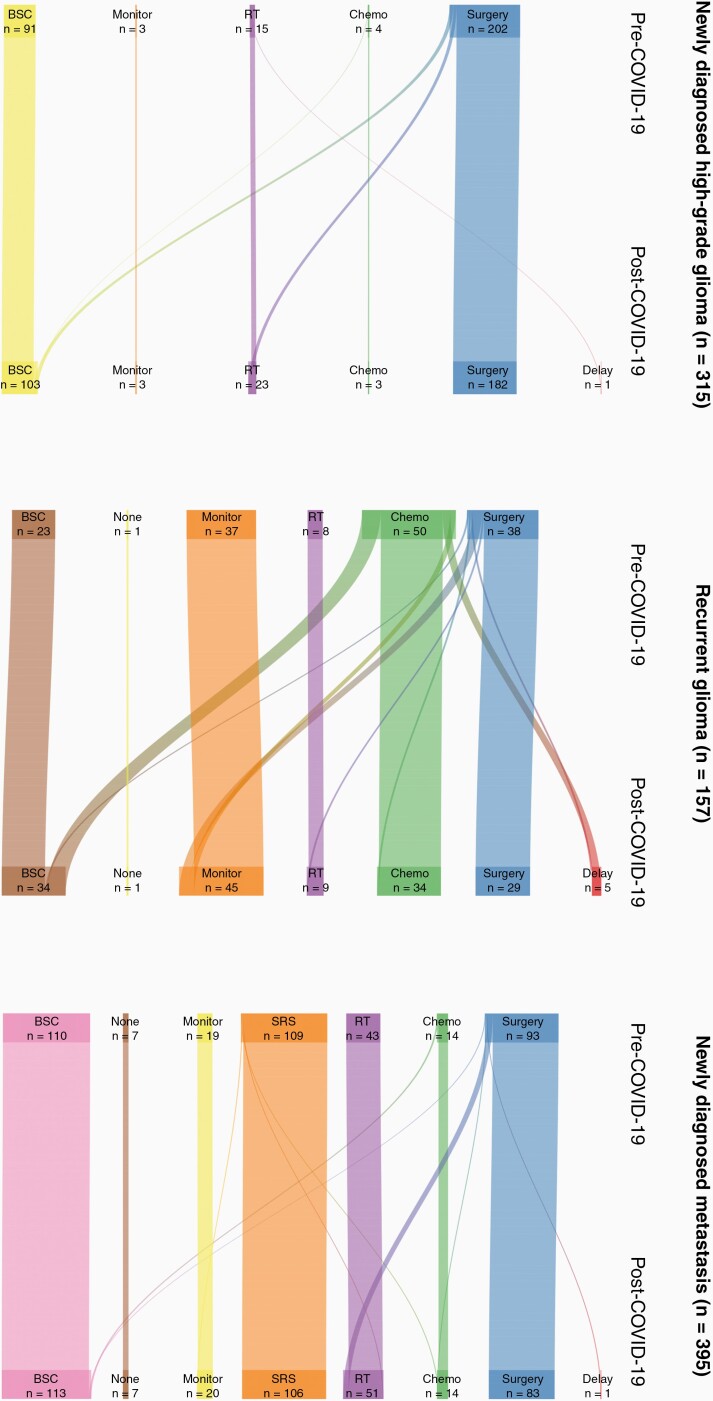
Sankey diagram of change in management decision as a result of the COVID-19 pandemic for patients with newly diagnosed high-grade glioma (*n* = 315), recurrent glioma (*n* = 157), and newly diagnosed metastasis (*n* = 395). Delay = delay or defer treatment, Chemo = chemotherapy, RT = radiotherapy, SRS = stereotactic radiosurgery, Monitor = interval monitoring, None = no treatment required, BSC = best supportive care.

Including all fifteen neuro-oncology units, 315 patients were referred with suspected high-grade glioma. The pre-COVID-19 and post-COVID-19 MDT decisions are provided in the Supplementary Material (Figure 3). Overall, 23 (7%) patients with newly diagnosed high-grade glioma had a change in management as a result of COVID-19. Comparing with those without a change of management, patients offered an alternative management were more likely to be ECOG 2 (*P* = .017) but there was no difference in age (*P* = .6) or sex (*P* = .2). Of the 202 patients who would have been offered surgery as “usual care” before COVID-19, 11 (5%) were instead offered best supportive care, and 9 (4%) were offered fractionated radiotherapy without the need for a diagnostic biopsy. Of all 157 patients referred with recurrent glioma, 26 (17%) had a change in MDT decision because of COVID-19. There was no significant difference in age (*P* = .2), sex (*P* = .7), or ECOG (*P* = .8) in the cohort of patients where a change in management plan was made. Of the 50 patients who would have been offered chemotherapy, 10 (20%) were instead offered best supportive care and a further 5 (10%) were recommended a delay in treatment. Further data is provided in the Supplementary Material (Figure 3).

Patients with newly diagnosed high-grade glioma suspected on MRI who subsequently underwent surgery revealed 10 grade III gliomas, and 153 glioblastomas, while patients presenting with recurrence revealed 1 recurrent grade III glioma and 16 recurrent GBM. Adjuvant treatment data for 162 high-grade glioma patients following surgery was available up to June 30, 2020 (Supplementary Table S3). Of these, 54 (33%) were treated with adjuvant chemoradiotherapy, 12 (7%) with chemotherapy alone, 38 (23%) with radiotherapy alone, and 58 (36%) were treated with surgery without additional oncological treatment.

During the study period, of all 445 high-grade glioma patients (based on MRI or confirmed high-grade glioma after surgery), there were 25 (5.6%) deaths reported. Three deaths occurred within 30 days of surgery (on days 3, 11, and 17).

### Management of Metastases

Including all 15 neuro-oncology units, 395 patients were referred with suspected cerebral metastases, of which, 314 (79%) were referred with a known primary cancer. MDT decisions are provided in the Supplementary Material (Figure 3). Fifteen (8%) patients with newly diagnosed metastasis had a change in management. There was no significant difference in age (*P* = .9), sex (*P* = .5), or ECOG (*P* = .5) in the cohort of patients where a change in management plan was made. Stereotactic radiosurgery was the most common pre-COVID-19 management plan (*n* = 109/395, 28%) whereas best supportive care was most common for post-COVID-19 management (*n* = 113/395, 29%). Including 89 cases of recurrent cerebral metastasis, data on oncological treatment was available for 484 patients (Supplementary Table 4). Of these, the majority of patients underwent a single treatment (SRS *n* = 103/132 78%, surgery *n* = 43/80 54%, radiotherapy *n* = 43/61 70%, chemotherapy *n* = 27/54 50%). The most common combination therapy was surgery and SRS (*n* = 14), followed by surgery and radiotherapy (*n* = 10). Patients with a recurrent metastasis were significantly more likely to receive chemotherapy for their systemic disease (21% vs. 9%, *P* = .002).

### Other Diagnoses

For patients with a radiological diagnosis of meningioma (*n* = 157, 17%), the MDT decision changed because of COVID-19 for 16 (10%) patients. The most common management plan for patients with suspected meningioma was for active monitoring (*n* = 84, 54%), followed by surgery (*n* = 50, 32%). Of those, 37 (74%) were recommended surgery post-COVID-19 with 9 (18%) patients recommended active monitoring and 4 (8%) patients recommended a delay in treatment. Within the study period, 30 (81%) patients with meningioma had successfully undergone surgery.

Results were similar for patients with newly diagnosed low-grade glioma (*n* = 60, 6%). MDT decisions changed due to COVID-19 for 10 (17%) patients. The most common pre-COVID-19 MDT plans were active monitoring (*n* = 29, 48%) and surgery (*n* = 28, 47%). Ultimately 19 patients with suspected low-grade glioma were offered surgery post-COVID-19, 7 (12%) were instead offered active monitoring and 2 (3%) patients were subject to a delay in planned treatment. Of available data for 18 patients, 16 had undergone surgery within the study period (89%).

### SARS-CoV-2 Infections

Suspected and confirmed SARS-CoV-2 infection data were available for 1184/1210 patients (98%). The overall infection rate was 2.4% (29/1184). Of the 28 patients where mortality data was available, 8 patients died (29%), with 5 deaths directly attributed to SARS-CoV-2 infection. Diagnosis of SARS-CoV-2 was made using a swab in 25 cases, whereas in 3 cases diagnosis was made radiologically and in one case it was unknown. Of the 348 patients undergoing surgery, 9 (2.6%) developed a confirmed SARS-CoV-2 infection (Supplementary Table S1). Eight cases were diagnosed preoperatively, and the rate of infection was not significantly different to the cohort of patients developing SARS-CoV-2 not undergoing an operation (*n* = 20/826, 2.4%, OR 1.1 95% CI 0.5–2.3, *P* = .852). Of the overall deaths in patients with high-grade glioma, 5 cases had a confirmed diagnosis of SARS-CoV-2, none of whom underwent surgery during the study period. In 3 of these cases, SARS-CoV-2 was documented as the primary cause of death.

## Discussion

### Change of MDT Recommendations During the COVID-19 Pandemic

This prospective, multicenter study reveals that during the height of the COVID-19 pandemic in the United Kingdom, a change in MDT decision making compared to “usual care” was recorded in 8.6% of cases. The reconfiguration of NHS services at the time included the decision on March 17, 2020 to postpone all non-urgent elective operation from April 15, 2020 at the latest. During the first weeks of the study coinciding with the onset of national lockdown and reconfiguration of NHS services in April 2020, the number of cases affected was as high as 19%. Two months later, while the lockdown and reconfiguration persisted, there were no affected cases in the final recorded MDTs. Patients with recurrent glioma were most affected by the pandemic, principally because chemotherapy was withheld. The rate of recorded SARS-CoV-2 infection during this period was low and was responsible for a small number of deaths recorded among patients with high-grade glioma.

Neuro-oncology services in the United Kingdom, similar to other oncology services, altered practice during the COVID pandemic. The results are similar to a national survey of MDT decision making performed in 18 neuro-oncology centers in the United Kingdom from 23rd March to 24th April 2020, where 10.7% of patients had their management changed as a result of the COVID-19 pandemic.^17^ A pan-cancer study by the UK Coronavirus Cancer Monitoring Project of 800 patients from 55 UK centers with a diagnosis of cancer (2% intracranial) showed that 22% of patients symptomatic of COVID-19 had a change of oncology management.^18^ In that cohort, management alterations were directly influenced by confirmed SARS-CoV-2 infection, whereas for our cohort, the suspicion of SARS-CoV-2 infection at the time of the MRI diagnosis was not significantly associated with change of management. The wider effects of the COVID-19 pandemic and perceived increased risk of death if patients were to become infected, are likely to have influenced decisions in our study.

Overall, 16% patients who would have been offered surgery as “usual care” were given a different recommendation and 24% patients who would have previously been offered chemotherapy had a change in recommendation. In the overall cohort we did not find that age or sex had a significant impact on MDT decision making. Although performance status was not associated with a change in management plan in the overall cohort, a poorer performance status did influence management recommendations in patients with a new diagnosis of a high-grade glioma. There was wide variation in the number of referrals received across units but this was due to a varying catchment population; all included units were offering regional services for patients with brain tumors.

There are 2 possible explanations to explain the reduction in changes in MDT decision making due to COVID-19 over the course of the study. It may be that the disruption to the neuro-oncology services caused by the COVID-19 pandemic was decreasing, or alternatively that neuro-oncology services adapted to provide a service despite COVID-19 restrictions. It is notable that of the oncology treatment reported, a third of patients who underwent an operation have not been treated with adjuvant chemotherapy or radiotherapy at the time of writing. Despite the reported pre-COVID-19 recommendation being surgery, 9% of patients with a radiologically defined, newly diagnosed high-grade glioma were offered best supportive care or fractionated radiotherapy without a tissue diagnosis.

### Surgical Management During the COVID-19 Pandemic

Comparing the practice observed to the published guidelines in March 2020, there has been a sustained delivery of surgical services for newly diagnosed high-grade glioma and metastasis with appropriate changes in MDT decisions to active monitoring for patients with low-grade glioma and meningioma.^8^ Of the 391 patients who were recommended surgery, 345 (88%) underwent their operation in the 2 months of greatest disruption due to COVID-19 in the UK. Furthermore, a very small minority (2.6%) of patients treated surgically developed SARS-CoV-2 and of these all but one was diagnosed preoperatively. This is a significant deviation from estimates and data published from the CovidSurg Collaborative where rates of cancellation in the 12 weeks of peak disruption were forecasted to be 37.7%, while rates of overall preoperative SARS-CoV-2 infection were 26% with a mortality rate of 18.4% for neurosurgical procedures (overall mortality 26%). In our study where mortality of patients with high-grade glioma was recorded, 3 patients died as a result of SARS-CoV-2 none of whom received surgical treatment. The data presented in this study is encouraging with regards to the continued delivery of surgical neuro-oncology services in the UK.^2,7^ This is particularly important given the key role of surgical resection.

While initial guidance from UK^8^ and international^9^ neuro-oncology experts has been published, it will be important to establish what overall treatment was provided to patients and perhaps consider a strategy for increasing the capacity of surgical neuro-oncology services and ease the pressures faced by local teams. Although nationally we managed to maintain surgical services for malignant brain tumors, a proportion of low-grade gliomas and meningioma were recommended for interval MRI follow-up rather than early surgery.

### Limitations

Our study has several limitations. Firstly, our primary outcome is based on a hypothetical question asked in the neuro-oncology MDTs on what their recommendation for management would have been prior to the COVID-19 pandemic. The exact location of the tumor and presenting symptoms used for priority stratification in guidelines subsequently published during the pandemic was not collected, so it was not possible to exactly compare practice to available guidelines. Therefore the generalizability of our findings is limited by context-specific factors during the COVID-19 pandemic, including healthcare provider resource utilization (including COVID-19 caseload) and staff sickness and subjective patient perception of safety in proceeding with admission to a hospital for treatment. Most of the change in management for newly diagnosed high-grade glioma occurred in older patients with poorer performance status, where we recommended for either best supportive care or radiotherapy (without tissue diagnosis). Pre-COVID many of these patients would have been offered surgery and radiotherapy +/- chemotherapy, despite the fact that there is often limited benefit from active oncology treatment in terms of overall survival.^19^ These data will continue to be collected in this study in preparation for a second report on the longer-term impact of COVID-19. Our patients had a minimum of only 30-days of follow up and it is likely that some patients may have gone on to have surgery outside of this follow-up window and are not captured in our analysis to date. These follow-up limitations are even more apparent when capturing the data of patients who did or did not receive chemotherapy or radiotherapy. This limitation will be mitigated with a planned second stage of data collection in July 2021 in order to measure this in detail and also to measure survival data. Thirdly, data on the provision of chemotherapy and radiotherapy may have been impaired by the inability to collect data for patients treated outside of their tertiary neuro-oncology center. Similarly, our study may underestimate the number of patients who contracted the COVID-19 virus during the study period—particularly if they contracted it in a community setting. A factor that we were not able to determine from this study was the change of referral volume to the neuro-oncology MDTs. Results from another unpublished survey of 30 UK neurosurgical units have shown a 27% reduction in the number of patients discussed in the neuro-oncology MDTs. Wide variations in referrals have been reported for other cancers, and a multicenter prospective study from centers in England and Northern Ireland showed that in April 2020, compared to prepandemic data, urgent referrals for early cancer diagnoses were down by 70–89%.^1^ A national report from Netherlands, reported an up to 26% reduction in cancer diagnosis and 60% reduction if skin cancer is included during the COVID-19 era.^20^

## Conclusions

Our study demonstrates that the COVID-19 pandemic had a noticeable impact on the management recommendations made by UK neuro-oncology specialists in the early stages of the COVID-19 pandemic. Delivery of first-line surgical treatment for newly diagnosed malignant tumors was maintained consistent with published national guidance with a very low rate of postoperative SARS-CoV-2. However, for patients with newly diagnosed malignant tumors there was notable disruption of chemotherapy treatments, in particular for patients with recurrent high-grade glioma. Further investigation is required into the impact of COVID-19 on the provision of chemotherapy, radiotherapy, and other non-surgical therapies and ultimately on patient outcomes and survival.

**Collaborators – British Neurosurgical Trainee Research Collaborative (BNTRC)**

**Table:** 

**Name**	**Affiliation**	**Contribution**
Yahia Al-Tamimi	Department of Neurosurgery, Sheffield Teaching Hospitals NHS Foundation Trust, Sheffield, UK	Acquisition of data, approval of manuscript
Andrew F. Alalade	Department of Neurosurgery, Royal Preston Hospital, Lancashire Teaching Hospital NHS Foundation Trust, Preston, United Kingdom	Acquisition of data, approval of manuscript
Erminia Albanese	Department of Neurosurgery, University Hospitals of North Midlands, Stoke-on-Trent, UK	Acquisition of data, approval of manuscript
Matthew Bailey	Manchester Centre for Clinical Neurosciences, Salford Royal NHS Foundation Trust, Salford, UK	Acquisition of data, approval of manuscript
Andrew R. Brodbelt	Department of Neurosurgery, The Walton Centre NHS Foundation Trust & University of Liverpool, Liverpool, UK	Acquisition of data, approval of manuscript
Anthony Chalmers	Institute of Cancer Sciences, University of Glasgow, UK	Acquisition of data, approval of manuscript
Huan Wee Chan	Department of Neurosurgery, University Hospital Southampton NHS Foundation Trust, Southampton, UK	Acquisition of data, approval of manuscript
David J. Coope	Manchester Centre for Clinical Neurosciences, Salford Royal NHS Foundation Trust, Salford, UK	Acquisition of data, approval of manuscript
Sarah Cundliffe	Manchester Centre for Clinical Neurosciences, Salford Royal NHS Foundation Trust, Salford, UK	Acquisition of data, approval of manuscript
Pietro I. D'Urso	Manchester Centre for Clinical Neurosciences, Salford Royal NHS Foundation Trust, Salford, UK	Acquisition of data, approval of manuscript
Helen Entwistle	Manchester Centre for Clinical Neurosciences, Salford Royal NHS Foundation Trust, Salford, UK	Acquisition of data, approval of manuscript
Rhiannon M. Evans	Velindre Cancer Centre, Cardiff, UK	Acquisition of data, approval of manuscript
Rebecca Fielding	Department of Neurosurgery, School of Medicine, Queen's Medical Centre, University of Nottingham, Nottingham, UK	Acquisition of data, approval of manuscript
Christos Gkolemis	Department of Neurosurgery, University Hospitals of North Midlands, Stoke-on-Trent, UK	Acquisition of data, approval of manuscript
Charlotte Hammerbeck-Ward	Manchester Centre for Clinical Neurosciences, Salford Royal NHS Foundation Trust, Salford, UK	Acquisition of data, approval of manuscript
D. Sanjeeva Jeyaretna	Department of Neurosurgery, John Radcliffe Hospital, Oxford, UK	Acquisition of data, approval of manuscript
Andrew T. King	Manchester Centre for Clinical Neurosciences, Salford Royal NHS Foundation Trust, Salford, UK	Acquisition of data, approval of manuscript
Raphael M. Laurente	Manchester Centre for Clinical Neurosciences, Salford Royal NHS Foundation Trust, Salford, UK	Acquisition of data, approval of manuscript
James Leggate	Manchester Centre for Clinical Neurosciences, Salford Royal NHS Foundation Trust, Salford, UK	Acquisition of data, approval of manuscript
Rachel Lewis	Department of Neurosurgery, The Royal London Hospital, Barts Health NHS Trust, London, UK	Acquisition of data, approval of manuscript
Jillian Maclean	Velindre Cancer Centre, Cardiff, UK	
Catherine McBain	The Christie NHS Foundation Trust, Manchester, UK	Acquisition of data, approval of manuscript
Grainne S. McKenna	Department of Neurosurgery, The Royal London Hospital, Barts Health NHS Trust, London, UK	Acquisition of data, approval of manuscript
Elizabeth Molloy	Manchester Centre for Clinical Neurosciences, Salford Royal NHS Foundation Trust, Salford, UK	Acquisition of data, approval of manuscript
Omar N. Pathmanaban	Manchester Centre for Clinical Neurosciences, Salford Royal NHS Foundation Trust, Salford, UK	Acquisition of data, approval of manuscript
Pradnya Patkar	Department of Neurosurgery, Lancashire Teaching Hospitals NHS Foundation Trust, Preston, UK	Acquisition of data, approval of manuscript
James Powell	Velindre Cancer Centre, Cardiff, UK	
Scott A. Rutherford	Manchester Centre for Clinical Neurosciences, Salford Royal NHS Foundation Trust, Salford, UK	Acquisition of data, approval of manuscript
Thomas Santarius	Division of Neurosurgery, Department of Clinical Neurosciences, Addenbrooke's Hospital & University of Cambridge, Cambridge, UK	Acquisition of data, approval of manuscript
Saurabh Sinha	Department of Neurosurgery, Sheffield Teaching Hospitals NHS Foundation Trust, Sheffield, UK	Acquisition of data, approval of manuscript
Murugan Sitaraman	Department of Neurosurgery, School of Medicine, Queen's Medical Centre, University of Nottingham, Nottingham, UK	Acquisition of data, approval of manuscript
Anna Solth	Department of Neurosurgery, Ninewells Hospital, Dundee, UK	Acquisition of data, approval of manuscript
Bhaskar Thakur	Department of Neurosurgery, The Royal London Hospital, Barts Health NHS Trust, London, UK	Acquisition of data, approval of manuscript
Andrea Wadeson	Manchester Centre for Clinical Neurosciences, Salford Royal NHS Foundation Trust, Salford, UK	Acquisition of data, approval of manuscript
Victoria Wykes	Department of Neurosurgery, Queen Elizabeth Hospital Birmingham, University Hospitals Birmingham NHS Foundation Trust, Birmingham, UK & Institute of Cancer and Genome Sciences, University of Birmingham, Birmingham, UK	Acquisition of data, approval of manuscript
Muhammed R. Zafar	Department of Neurosurgery, School of Medicine, Queen's Medical Centre, University of Nottingham, Nottingham, UK	Acquisition of data, approval of manuscript

## Supplementary Material

vdab014_suppl_Supplementary_Materials

## Notes

**Authorship Statement.** Conception and design: D.M.F., R.J.P., M.T.C.P., G.S., P.M.B., P.L.G., P.J.H., K.K., A.G.K., P.P., S.J.P., O.R., S.J.S., Su.S., S.T., J.Y.L., C.W., M.D.J. Acquisition of data: D.M.F., R.J.P., M.T.C.P., G.S., Y.A.C., F.C., M.T.H., T.E., F.G.E., M.H., N.J.M., C.P.M., I.P., S.J.P., O.R., W.S., Sy.S., S.J.S., I.L.S., S.T., J.Y.L., C.W., M.D.J. Analysis and interpretation of data: D.M.F., R.J.P., M.T.C.P., G.S., M.D.J. Writing, review and/or revision of manuscript: D.M.F., R.J.P., M.T.C.P., G.S., P.M.B., Y.A.C., F.C., M.T.H., T.E., F.G.E., P.L.G., M.H., P.J.H., K.K., A.G.K., N.J.M., C.P.M., I.P., P.P., S.J.P., O.R., W.S., Sy.S., S.J.S., I.L.S., Su.S., S.T., J.Y.L., C.W., M.D.J. All authors and collaborators listed were involved in patient identification, data collection, and reviewed the manuscript prior to submission.
