# Supplementary material for: CovidNeuroOnc: A UK multicenter, prospective cohort study of the impact of the COVID-19 pandemic on the neuro-oncology service
Source: Neurooncol Adv. 2021 Jan 28;3(1):vdab014. doi: 10.1093/noajnl/vdab014 (PMC7928638; doi:10.1093/noajnl/vdab014)
Supplement: vdab014_suppl_Supplementary_Materials [file vdab014_suppl_supplementary_materials.docx]

**Supplementary Material**

*Table S1 – Descriptive comparative characteristics of patients undergoing surgery for intracranial tumours (n=1198)*

| **Characteristic** | **No**, N = 844*^1^* | **Yes**, N = 354*^1^* | **p-value***^2^* |
| --- | --- | --- | --- |
| Age |  |  | <0.001 |
| 16-19 | 2 (0.2%) | 3 (0.8%) |  |
| 20-29 | 18 (2.1%) | 17 (4.8%) |  |
| 30-39 | 45 (5.3%) | 20 (5.6%) |  |
| 40-49 | 71 (8.4%) | 37 (10%) |  |
| 50-59 | 157 (19%) | 97 (27%) |  |
| 60-69 | 199 (24%) | 105 (30%) |  |
| 70-79 | 225 (27%) | 67 (19%) |  |
| 80-89 | 115 (14%) | 8 (2.3%) |  |
| 90+ | 12 (1.4%) | 0 (0%) |  |
| Sex |  |  | 0.078 |
| Female | 447 (53%) | 167 (47%) |  |
| Male | 397 (47%) | 187 (53%) |  |
| ECOG |  |  | <0.001 |
| 0 | 296 (36%) | 159 (45%) |  |
| 1 | 265 (32%) | 130 (37%) |  |
| 2 | 151 (18%) | 53 (15%) |  |
| 3 | 94 (11%) | 8 (2.3%) |  |
| 4 | 25 (3.0%) | 4 (1.1%) |  |
| Presentation |  |  | <0.001 |
| New diagnosis | 628 (74%) | 313 (88%) |  |
| Recurrence | 216 (26%) | 41 (12%) |  |
| New diagnosis - radiological diagnosis |  |  | <0.001 |
| High-grade glioma | 134 (21%) | 161 (51%) |  |
| Low-grade glioma | 38 (6.1%) | 19 (6.1%) |  |
| Meningioma | 126 (20%) | 30 (9.6%) |  |
| Metastasis | 269 (43%) | 72 (23%) |  |
| Other | 32 (5.1%) | 8 (2.6%) |  |
| Primary CNS lymphoma | 18 (2.9%) | 21 (6.7%) |  |
| Vestibular schwannoma | 9 (1.4%) | 2 (0.6%) |  |
| Recurrence – original histopathology |  |  | 0.2 |
| Glioma | 108 (50%) | 21 (51%) |  |
| Meningioma | 24 (11%) | 2 (4.9%) |  |
| Metastasis | 61 (28%) | 10 (24%) |  |
| Other | 15 (6.9%) | 7 (17%) |  |
| Primary CNS Lymphoma | 2 (0.9%) | 1 (2.4%) |  |
| Vestibular schwannoma | 6 (2.8%) | 0 (0%) |  |
| SARS-CoV-2 suspected at time of MRI diagnosis | 22 (2.6%) | 8 (2.3%) | 0.9 |
| SARS-CoV-2 suspected at time of surgery | 5 (0.6%) | 6 (1.7%) | 0.093 |
| Confirmed SARS-CoV-2 infection | 20 (2.4%) | 9 (2.6%) | >0.9 |
| *^1^*Statistics presented: n (%)  *^2^*Statistical tests performed: chi-square test of independence; Fisher's exact test | | | |

*Table S2 – Histopathological characteristics of patients following surgery for intracranial tumours (n=354)*

| **Characteristic** | **N = 354***^1^* |
| --- | --- |
| Surgery offered |  |
| Biopsy | 82 (24%) |
| Resection | 258 (76%) |
| Surgery performed |  |
| Biopsy | 82 (24%) |
| Gross-total resection | 180 (52%) |
| Sub-total resection | 81 (24%) |
| Histopathological diagnosis |  |
| Glioma | 193 (56%) |
| Meningioma | 34 (9.9%) |
| Metastasis | 69 (20%) |
| Other | 28 (8.1%) |
| Primary CNS Lymphoma | 18 (5.2%) |
| Vestibular schwannoma | 2 (0.6%) |
| Glioma grade |  |
| 2 | 17 (9.0%) |
| 3 | 11 (5.8%) |
| 4 | 161 (85%) |
| Glioma type |  |
| Anaplastic astrocytoma | 8 (4.3%) |
| Anaplastic oligodendroglioma | 1 (0.5%) |
| Astrocytoma | 17 (9.1%) |
| Glioblastoma | 155 (83%) |
| Oligodendroglioma | 6 (3.2%) |
| IDH status |  |
| Mutated | 32 (17%) |
| Wild-type | 140 (76%) |
| MGMT status |  |
| Methylated | 61 (36%) |
| Unmethylated | 62 (37%) |
| 1q19q status |  |
| Deleted | 8 (4.4%) |
| Intact | 12 (6.6%) |
| Meningioma grade |  |
| 1 | 20 (61%) |
| 2 | 12 (36%) |
| 3 | 1 (3.0%) |
| Metastasis primary |  |
| Breast | 6 (8.7%) |
| Lung | 31 (45%) |
| Melanoma | 7 (10%) |
| Other | 20 (29%) |
| Renal | 5 (7.2%) |
| *^1^*Statistics presented: n (%) | |

*Table S3 – Oncological treatment characteristics for patients with histologically confirmed grade 3 and 4 glioma following surgical treatment (n=162), and patients with suspected newly diagnosed or recurrent metastasis (n=484)*

| **Characteristic** | **High-grade glioma N=162** | | | **Metastasis N=484** | | |
| --- | --- | --- | --- | --- | --- | --- |
|  | **New diagnosis**,  N = 145*^1^* | **Recurrence**, N = 17*^1^* | **p-value***^2^* | **New diagnosis**, N = 395*^1^* | **Recurrence**, N = 89*^1^* | **p-value***^2^* |
| Surgery offered |  |  | 0.076 |  |  | >0.9 |
| Biopsy | 37 (26%) | 1 (5.9%) |  | 7 (8.4%) | 0 (0%) |  |
| Resection | 106 (74%) | 16 (94%) |  | 76 (92%) | 8 (100%) |  |
| Surgery performed |  |  | 0.2 |  |  | 0.7 |
| Biopsy | 34 (24%) | 1 (5.9%) |  | 5 (6.8%) | 0 (0%) |  |
| Gross-total resection | 59 (41%) | 8 (47%) |  | 63 (86%) | 6 (86%) |  |
| Sub-total resection | 50 (35%) | 8 (47%) |  | 5 (6.8%) | 1 (14%) |  |
| Radiotherapy | 89 (61%) | 3 (18%) | 0.001 | 103 (26%) | 29 (33%) | 0.3 |
| Chemotherapy | 59 (41%) | 7 (41%) | >0.9 | 52 (13%) | 9 (10%) | 0.5 |
| *^1^*Statistics presented: n (%)  *^2^*Statistical tests performed: Fisher's exact test; chi-square test of independence | | | | | | |
